# To Purify Water

**Published:** 1857-12

**Authors:** 


### TO PURIFY WATER.

"Put alum in it." Oh, yes, certainly! This silly article has been going the round of the scissorsing newspapers for months. No doubt, many persons who have tried it have regarded it as a beautiful and simple fact. Yes! the fact is as simple as the theory educed therefrom. We have been always under the impression, that to purify anything, something must be taken from it. But to purify dirty water, we must give it a dose of physic—alum being a speedy and certain emetic. It strikes us that, if water is dirty, the best use of it is to throw it away, in a land where there is a spring or stream almost in sight of every door.

The alum will throw the dirt to the bottom, but more or less of it remains in the water. But the alum is a mineral medicine causing active purging and vomiting, and gradually undermining the digestive functions by its astringent, puckering qualities. On the other hand, the sediment which is ordinarily found in water will settle itself, if allowed time enough; besides, it is almost wholly of vegetable origin, and less hurtful than the amount of alum requisite to throw it to the bottom.

